# The Role of Unexpected Infection in Acetabular Erosion After Hip Hemiarthroplasty

**DOI:** 10.3390/medicina61122141

**Published:** 2025-11-30

**Authors:** Luis-Rodrigo Merino-Rueda, Ricardo Fernández-Fernández, Eduardo García-Rey

**Affiliations:** 1 Department of Orthopedic Surgery and Traumatology, La Paz University Hospital, 28046 Madrid, Spain; luisrodrigo.merino@salud.madrid.org (L.-R.M.-R.); ricardo.fernandez@salud.madrid.org (R.F.-F.); 2 Faculty of Medicine, Autonomous University of Madrid, C. Arzobispo Morcillo, 4, Fuencarral-El Pardo, 28029 Madrid, Spain

**Keywords:** acetabular erosion, hemiarthroplasty, low-grade infection, femoral neck fracture, conversion surgery

## Abstract

*Background and objectives*: Hemiarthroplasty (HA) remains one of the most common treatments for displaced femoral neck fractures in the elderly, providing pain relief, early mobilization and a low reoperation risk. Acetabular erosion is a recognized late complication of this procedure, but early cartilage wear and its potential relationship with infection remain poorly understood. The aim of this study was to describe the clinical and microbiological characteristics of patients who required conversion to total hip arthroplasty (THA) because of acetabular erosion and to analyze the possible role of unexpected infection as a contributing factor. *Materials and methods*: A retrospective observational study was performed including all patients treated between 2007 and 2019 who underwent conversion of a failed HA to THA due to acetabular erosion after femoral neck fracture. Microbiological analysis was performed in all cases through multiple intraoperative samples. Patients were classified into two groups, with and without infection, according to positive microbiological cultures. *Results*: Forty-four patients were included, with a median age of 80.5 years (74–85). The median time to acetabular erosion was 25.4 months (10.4–47.4). Infection was identified in six patients (13.6%), all within the first six months after fracture (*p* = 0.029). The median time to erosion was shorter in infected patients (4.0 versus 29.8 months, *p* < 0.001). No other demographic, functional, or implant-related variables were associated with infection. There were three re-revisions, two due to recurrent dislocation and one periprosthetic infection in a hip without unexcepted positive culture. All patients with positive intraoperative culture were successfully managed with antibiotherapy. Postoperative functional scores improved significantly in both groups. Fifteen patients (34.1%) died during follow-up. *Conclusions*: Early acetabular erosion after hemiarthroplasty may represent a manifestation of previously unrecognized low-grade infection, particularly in frail elderly patients. Despite advanced age and multiple comorbidities, conversion to THA achieved significant functional improvement with an acceptable complication rate. Prospective studies with larger populations are warranted to confirm the relationship between infection and early acetabular cartilage loss.

## 1. Introduction

Hip fractures represent one of the most devastating injuries in the elderly population, with projections indicating a continued rise, imposing not only a substantial individual burden but also a major socioeconomic cost worldwide [1]. Hemiarthroplasty (HA) remains one of the most frequently performed procedures for displaced femoral neck fractures in frail and elderly patients, offering pain relief and early mobilization with relatively low operative risk compared to total hip arthroplasty (THA) [1,2].

Acetabular erosion is a recognized late complication after HA. It refers to progressive degeneration of the acetabular cartilage and subsequent bone loss caused by abnormal mechanical interaction between the prosthetic head and the native acetabulum [3,4]. The etiology of acetabular erosion remains multifactorial and incompletely understood. Reported contributing factors include patient-related variables such as biological age, activity level, fragility, and comorbidities, as well as surgical and implant-related aspects such as femoral head size, limb length discrepancy, implant design (either unipolar or bipolar), and proximal femoral morphology [2,4,5,6,7,8]. While some studies report higher rates of erosion in unipolar designs [2], others have documented that up to 66% of patients may develop radiographic acetabular changes over time even with bipolar implants [4]. Some studies suggest that the incidence is higher among patients younger than 75 years, potentially due to greater functional demand and higher mechanical loading on the acetabular surface [4]. Nonetheless, other studies do not provide conclusive evidence in this regard, and the available data remain heterogeneous, often stemming from small case series and retrospective observational designs. Additionally, large-scale retrospective analyses indicate that the overall incidence of symptomatic acetabular erosion is low, with only a minority of cases ultimately requiring conversion to THA [6].

Several studies have reported that the time to onset of acetabular erosion after hemiarthroplasty is highly variable. Some authors suggest that very early presentation may be influenced by the pre-existing condition of the acetabular cartilage, indicating that cartilage status at the time of the index procedure could play a role in the early development of erosion [6]. Other studies have highlighted implant- and anatomy-related factors, such as the use of unipolar prostheses [2], cylindrical femoral morphology [5], smaller femoral head sizes, limb length discrepancy, and reduced bone density, as contributors to earlier development of erosion [6]. These findings indicate that both surgical variables and patient-specific factors such as bone quality and overall fragility may determine the timing and risk of acetabular cartilage wear. Infection has also been identified as a relevant trigger for accelerated erosion and acetabular protrusion in certain contexts. Case reports and small series have described chronic or low-grade infection associated with intrapelvic protrusion following HA or THA [9,10].

Low-grade infection may accelerate acetabular cartilage deterioration through persistent synovial inflammation, release of catabolic cytokines and matrix metalloproteinases, altered synovial fluid composition, and increased joint friction. These mechanisms can disrupt cartilage homeostasis and promote early erosion, even in the absence of overt clinical signs of infection, thereby supporting the relevance of exploring this potential association [11,12]. The role of infection in the development of acetabular erosion after HA remains insufficiently understood. In this study, we aimed to evaluate the baseline characteristics of patients undergoing conversion from HA to THA due to acetabular erosion, with particular attention to the possible contribution of an unexpected infection as a factor underlying cartilage damage.

## 2. Materials and Methods

### 2.1. Study Design and Patient Population

This is a retrospective observational cohort study. After approval by the local ethics committee (PI-2626), we included all patients treated at a single center between January 2007 and December 2019 who underwent conversion of a failed HA to THA due to acetabular erosion following femoral neck fracture. The study was based on data obtained from medical records, surgical protocols, and imaging studies. Inclusion criteria were: (a) patients treated during the study period with conversion from HA to THA for acetabular erosion, and (b) availability of intraoperative microbiological samples for culture. Exclusion criteria were: (a) absence of intraoperative samples for microbiological cultures, and (b) insufficient clinical, radiological, or surgical information in the medical records. A total of 53 patients met the initial screening criteria; 9 were excluded (7 due to missing or incomplete intraoperative microbiological sampling and 2 due to insufficient clinical data), leaving 44 patients for the final analysis.

Demographic variables, including sex, side, body mass index (BMI), and age at fracture diagnosis, were collected and analyzed along with clinical data and pre-fracture functional status. Patient mobility was assessed with the Functional Ambulation Classification (FAC), which ranges from 0 (inability to walk) to 5 (complete independence in any environment); scores of 1 and 2 indicate different levels of dependence on assistance or supervision, while scores of 3 to 5 reflect increasing independence [13]. Disability was measured using the Barthel Index (0 = total dependence, 100 = total independence) [14]. For analysis, patients were classified as independent in basic activities of daily living (BADL) if their score was ≥91, and as dependent otherwise [14,15]. Cognitive function was evaluated with the Pfeiffer scale, a 10-item questionnaire assessing orientation, memory, and calculation, in which impairment is classified according to the number of errors, allowing rapid detection of cognitive decline or dementia [16]. Global mental disability was assessed using the Red Cross Mental Disability Scale (RCMDS), which evaluates cognitive impairment on a 0–5 scale—ranging from normal function (0) to maximum dependence (5)—based on memory, orientation, and communication abilities. This brief clinician-administered tool is widely used in geriatric settings to screen global cognitive and functional mental status in frail, homebound, or institutionalized elderly populations [17]. The main comorbidities were recorded, including hypertension, diabetes mellitus, chronic kidney disease, rheumatoid arthritis, and immunosuppression (either pharmacological or disease-related). Polypharmacy was also assessed, defined as the regular concomitant use of five or more medications per day, excluding nutritional supplements [18]. HA implant recorded data included femoral stem fixation and head type (monopolar or bipolar).

### 2.2. Surgical and Postoperative Management

Acetabular erosion was defined as the progressive loss of acetabular cartilage on anteroposterior pelvic radiographs, accompanied by increasing groin pain and functional limitation [4]. Acetabular defects were classified according to Paprosky, which categorizes bone loss based on the integrity of the acetabular walls, dome, and columns. Type 2 defects present moderate bone loss with preservation of the acetabular rim and both columns, allowing stable cup support (subtypes 2A: superior bone loss; 2B: superolateral migration; 2C: medial wall involvement). Type 3 defects indicate severe bone loss with compromised structural support, where the hip center is displaced and the acetabular columns are weakened (3A: substantial superior migration; 3B: massive bone loss with pelvic discontinuity risk) [19].

Time to acetabular erosion was defined as the interval between HA and the appearance of the first clinical or radiographic sign of erosion. Time to conversion surgery was defined as the interval between the diagnosis of acetabular erosion and conversion to THA.

For microbiological assessment, five intraoperative tissue samples were collected in accordance with our institutional revision-surgery protocol, which mandates obtaining these specimens before administering any antibiotics. Standard perioperative prophylaxis was given only after sample collection: all patients received a single dose of cefazolin 2 g, except for two individuals with beta-lactam allergy, who received vancomycin 2 g instead.

Regarding preoperative evaluation, laboratory tests (including inflammatory markers), targeted microbiological aspiration, or imaging studies specifically aimed at ruling out infection were not routinely performed, as no patient presented clinical suspicion of infection prior to surgery. Consequently, the positive cultures identified during revision should be considered incidental findings arising from this systematic intraoperative sampling protocol.

Acetabular reconstruction depended on bone defect. Small and moderate bone defects were reconstructed using either a cemented cup or a cementless press-fit cup, depending on bone quality and intraoperative stability. In those hips with larger defects, we performed a bone impaction grafting and implanted a cemented cup, using metallic meshes or trabecular metal augments if a segmental bone defect was present. In all cases, the femoral stem was left in place. Surgical parameters documented included the surgical approach, cup characteristics (size, model, fixation method—cemented or press-fit with/without screws—and standard vs. dual-mobility design), femoral head size, and intraoperative complications.

Infection was diagnosed according to the Musculoskeletal Infection Society (MSIS) criteria. As this study focused exclusively on intraoperative microbiological assessment and no preoperative suspicion of infection existed, only the intraoperative major MSIS criterion was applicable. In this context, infection was defined as the isolation of the same microorganism with an identical antimicrobial susceptibility profile (indistinguishable antibiogram) in at least two intraoperative specimens, fulfilling one of the two major MSIS criteria [20]. All patients diagnosed with an unexpected infection were systematically assessed by the Infectious Diseases team for treatment and follow-up. All patients received targeted antibiotic therapy guided by antibiogram results and current clinical practice guidelines, starting with an initial intravenous regimen for 2–3 weeks followed by oral therapy to complete a total course of 6–12 weeks. Antibiotics were continued until clinical resolution and normalization of inflammatory biomarkers, such as C-reactive protein and erythrocyte sedimentation rate.

### 2.3. Clinical and Radiological Follow-Up

Patients were evaluated annually throughout follow-up. Functional outcomes and quality of life were assessed using the self-reported modified Harris Hip Score (mHHS) [21], which rates hip pain and function on a 0–100 scale, with higher scores indicating better clinical status. Assessments were performed at the preoperative visit upon inclusion on the surgical waiting list and at the final follow-up. Radiographic analysis was performed on standardized anteroposterior (AP) pelvic radiographs, with magnification corrected using the diameter of the femoral head. All radiographs were evaluated by a single senior surgeon. Measured parameters included the acetabular inclination angle, horizontal distance to Köhler’s line, vertical distance to the teardrop base, the center of rotation distance according to Ranawat’s method [19,22], assessed on both the immediate postoperative and the last available follow-up radiographs.

The sample was stratified into two groups, with and without infection, to evaluate demographic, clinical, and HA-related variables potentially associated with an increased risk of infection.

### 2.4. Statistical Analysis

Categorical variables were expressed as counts and percentages, and quantitative variables as mean ± standard deviation, or median [interquartile range], based on their distribution. Group comparisons for categorical variables were conducted using the chi-squared test or the Fischer’s exact test as appropriate, while the Wilcoxon rank-sum test was performed as a non-parametric test for continuous variables, given the overall sample size. A *p*-value < 0.05 was considered statistically significant and all the analyses were performed in Stata BE 18.0 (StataCorp, College Station, TX, USA).

## 3. Results

Of the 53 patients who underwent prosthetic conversion during the study period, 44 were included after application of the exclusion criteria. The median age at fracture diagnosis was 80.5 years (74–85). The cohort was predominantly female (n = 35, 79.6%), with 26 cases (59.1%) involving the left hip. The median body mass index (BMI) was 26 (25–26). All patients had a Functional Ambulation Classification (FAC) score ≥ 3, and the median Barthel Index was 95 (85–95). Demographic and baseline characteristics of the study population are summarized in Table 1.

Regarding the type of HA, 25 cases (56.8%) were cemented and 19 (43.2%) cementless. The most frequently brands were CS-Plus^®^ (Smith & Nephew, London, UK) in 21 cases (47.7%) and SL-Plus^®^ (Smith & Nephew, London, UK) in 19 cases (43.2%). The characteristics of the HA included in the study are shown in Table 2.

The median time to acetabular erosion was 25.4 months (10.4–47.4), while the median time to conversion surgery was 43.2 months (20.5–61.2). The median age at the time of conversion surgery was 81.8 years (77.5–88.8). The median follow-up was 98.1 months (63.5–153.5). The surgical approach was lateral in 10 cases (22.7%) and posterolateral in 34 (77.3%). The distribution of acetabular defects according to the Paprosky classification was: type 2A, 12 cases (27.3%); type 2B, 6 (13.6%); type 2C, 1 (2.3%); type 3A, 21 (47.7%); and type 3B, 4 (9.1%). For bone defect reconstruction, bone impaction grafting was used in 29 patients (65.9%), mesh reconstruction in 7 (15.9%), and a tantalum supplement in 1 (2.3%).

The median implanted cup size was 50 mm (48–50), with 48 mm (n = 17, 38.6%) and 50 mm (n = 16, 36.4%) being the most frequent. Regarding fixation, 31 cups (59.1%) were cemented. The most frequently used model was Rimfit^®^ (Stryker, Kalamazoo, MI, USA), used in 23 patients (52.3%). Supplementary fixation with two screws was performed in 10 of the 18 uncemented cups. A dual mobility device was implanted in 4 patients (9.1%). Two intraoperative complications (4.6%) were recorded one posterior column fracture treated with open reduction and plate fixation and one medial wall fracture managed with impacted graft and a cemented cup. Table 3 shows the variables related to acetabular reconstruction.

Intraoperative cultures were positive in 6 cases (13.6%). Isolated organisms included methicillin-sensitive *Staphylococcus epidermidis* (n = 2), methicillin-resistant *Staphylococcus epidermidis* (n = 1), *Staphylococcus hominis* (n = 1), *Escherichia coli* (n = 1), and *Pseudomonas aeruginosa* (n = 1). All patients received antibiotic therapy as guided by the Infectious Diseases team. Management consisted of an initial course of targeted intravenous antibiotics for 2–3 weeks, followed by oral consolidation therapy for 6–12 weeks, adjusted according to clinical evolution and normalization of inflammatory biomarkers.

The potential association between baseline patient characteristics, type of hemiarthroplasty, and risk of infection was analyzed, with detailed results presented in Table 4. No significant differences were found for age at fracture diagnosis, sex, BMI, FAC, Barthel Index, Pfeiffer scale, Red Cross Mental Disability Scale, BADL, comorbidities, or polypharmacy. Similarly, no differences were observed according to the type of HA, including prosthesis model, head size, or mono- versus bipolar design. Infection occurred exclusively in patients with cemented stems, showing a significant association with the fixation method (*p* = 0.029).

Time to acetabular erosion was also significantly shorter in patients with infection, with a median of 4 months (3.7–4.6) compared to 29.8 months (14.8–50.7) in those without a positive microbiological culture (*p* < 0.001). In contrast, no significant differences were found in time to conversion surgery.

During follow-up, no significant differences in postoperative complications were observed between groups. Overall, complications occurred in 9 patients (20.5%). There were 6 cases of dislocation: four isolated episodes managed conservatively with closed reduction, rest, and physiotherapy, and two recurrent dislocations treated surgically—one with cup replacement using a dual mobility system and the other with a constrained liner. No differences were found in acetabular component orientation or position when comparing immediate postoperative radiographs with those at the final follow-up. Two cases of aseptic loosening were diagnosed late in elderly patients with very low functional demand—one in each group—both managed conservatively without surgery due to their limited functional expectations. One postoperative infection was treated with a two-stage revision. Among patients with infection, there was only one case of dislocation. The single postoperative infection occurred in a patient without positive intraoperative cultures.

Compared with baseline, the median mHHS improved similarly in both groups. Fifteen patients (34.1%) died during follow-up: three in the infection group and twelve in the aseptic erosion group (*p* = 0.394). Table 5 summarizes the clinical and radiological variables related to follow-up in both groups.

## 4. Discussion

Although HA remains the most common surgical treatment for displaced femoral neck fractures in the elderly, a subset of patients may develop early acetabular cartilage wear, leading to pain, functional decline, and the eventual need for conversion to total hip arthroplasty [1,6]. Despite being a recognized late complication, early acetabular erosion continues to represent a diagnostic and therapeutic challenge. In our study, the possibility of infection emerged as a factor that may warrant consideration in cases of early acetabular erosion.

### 4.1. Patient- and Implant-Related Factors

Patients undergoing HA constitute a heterogeneous population with wide variability in comorbidity burden, frailty status, and functional capacity, which may influence the development of acetabular erosion [4,6]. Previous studies have highlighted the contribution of both mechanical and patient-related factors, including implant design, head diameter, activity level, and overall bone quality [3,4]. Patient-related factors such as younger age and higher activity levels may also contribute to increased mechanical stress on the acetabular surface. Macheras et al. reported that even among elderly individuals, those under 75 years exhibited higher rates of acetabular erosion, likely reflecting greater physical activity and mechanical demand. In contrast, more frail patients tended to show lower rates of symptomatic wear [4]. Similarly, Mahmoud et al. and Theil et al. emphasized that functional activity, rather than chronological age, is the main determinant of acetabular cartilage degeneration [6,8]. In our cohort, although the median age at fracture was 80.5 years [74.0–85.0] and no differences were found in comorbidities or frailty-related variables, all patients who developed acetabular erosion were ambulatory and functionally independent (FAC ≥ 3), consistent with these findings. This preserved functional capacity implies higher mechanical loading and supports considering conversion to total hip arthroplasty when symptoms persist, as even modest improvements in pain and mobility can meaningfully enhance quality of life. Other variables, including bone mineral density, proximal femoral morphology, and fixation method, have been discussed as contributing but non-independent factors [8,23]. However, despite extensive literature examining mechanical and demographic contributors, no prior studies have clearly established a direct association between these factors and the early onset of acetabular erosion. In this context, our work explores the possible role of infection in patients already diagnosed with acetabular erosion, suggesting it as a potential and previously under-recognized mechanism that may contribute to early cartilage damage.

### 4.2. Unexpected Infection and Early Erosion

When comparing our findings with those of previous studies, the incidence of infection and the timing of acetabular erosion appear as potential differentiating factors that may help contextualize the early presentation observed in some patients. Several published case reports and small series have described rapid acetabular chondrolysis, protrusion, with chronic or low-grade infection after HA. Stiehl et al. reviewed 16 cases of prosthetic protrusion and found that 11 were associated with chronic infection [9]. Similarly, Lim et al. reported a case of protrusion after bipolar HA in which chronic periprosthetic joint infection was the primary cause [10]. In the setting of very early erosions, infection has also been suggested as a possible contributor: Adenikinju et al. (2019) described a case of rapid acetabular chondrolysis occurring within weeks of HA, in which the authors recommended ruling out periprosthetic joint infection as an underlying etiology [24]. The median time to erosion in our cohort (25.4 months) was consistent with previous reports [6], yet patients with unexpected infection presented significantly earlier erosion (median 4 months), aligning with case-based evidence describing rapid chondrolysis or early protrusion potentially secondary to infection [10,24]. In our series, all infection-related erosions occurred within the first six months after the index fracture, supporting the hypothesis that early cartilage loss may be a radiological manifestation of unrecognized low-grade infection. Although frailty-related variables did not reach statistical significance, infected patients showed a shorter median time to erosion and appeared overall more clinically fragile, raising the possibility that systemic vulnerability may facilitate infection persistence or delayed detection. Additionally, all patients with infection had cemented stems; this distribution likely reflects selection bias, as cemented implants are preferentially used in frailer individuals with greater comorbidity burden. Nevertheless, given the very small number of infected cases and the limited sample size, these findings must be interpreted cautiously and cannot be generalized.

With respect to pathogen-specific differences, our cohort included low-virulence skin commensals such as Staphylococcus epidermidis (methicillin-sensitive and methicillin-resistant) and Staphylococcus hominis, as well as Gram-negative organisms such as Escherichia coli and Pseudomonas aeruginosa. Low-virulence coagulase-negative staphylococci are well known for their capacity to produce biofilm and cause indolent infections with minimal systemic response, making them difficult to distinguish from contaminants. For this reason, we adhered strictly to MSIS criteria, requiring two concordant positive cultures with identical antibiograms to minimize false positives. In contrast, Gram-negative organisms are less commonly described in unexpected PJI and may reflect different pathogen reservoirs or perioperative contamination patterns. Their presence may also imply a more aggressive biological behavior, but due to the small sample size, no definitive conclusions can be drawn regarding pathogen-specific impact on the severity or timing of erosion [20,25]. Although our cohort does not permit statistical comparison between microbiological subgroups, these findings collectively emphasize that both low-virulence skin flora and Gram-negative organisms may contribute to early cartilage damage when unrecognized. Future multicenter studies with larger samples could clarify whether specific pathogens confer distinct risks or clinical trajectories.

### 4.3. Antibiotic Management and Rationale

Our antibiotic management for patients with unexpected positive intraoperative cultures aligns closely with contemporary recommendations for low-grade PJI. Current literature emphasizes that optimal treatment of prosthetic joint infections requires a combination of adequate surgical management and prolonged, pathogen-directed antibiotic therapy, typically beginning with a high-dose intravenous phase followed by an oral consolidation regimen [25]. In our cohort, all patients received targeted intravenous therapy for 2–3 weeks, followed by an oral regimen for an additional 6–12 weeks under the supervision of an Infectious Diseases specialist. This management strategy aligns with guideline-based recommendations that emphasize an initial high-dose intravenous phase to achieve rapid bactericidal activity in low-virulence organisms, followed by a prolonged oral course to enhance biofilm penetration and reduce the risk of relapse. Although unexpected positive cultures after HA conversion represent a different scenario from classical PJI, our protocol reflects established principles for the treatment of chronic or indolent infections, providing a sound therapeutic rationale for the favorable outcomes observed in our series, with no cases of loosening or reinfection among the six affected patients.

### 4.4. Functional Recovery and Outcomes After Conversion to THA

Functional recovery following conversion to THA was satisfactory in our cohort, with a median mHHS improvement comparable to that reported in previous meta-analyses [7]. Poursalehian et al. observed a mean improvement of 39 points in mHHS after conversion, with complication rates between 8–10%. In our study, the overall complication rate was 20.5%, within the reported range for conversion arthroplasty [7], although only three cases required surgical management. Of the 44 cups, only three showed late failure, and just one required revision due to infection. The remaining two cases, both cemented, were managed conservatively because of the patients’ low functional demand at the time of complication diagnosis (FAC = 1). The case that developed a late infection treated with a two-stage revision belonged to the aseptic erosion group. These findings support the notion that, in elderly patients with low functional demand, radiographic loosening without associated pain or instability may not justify reintervention [4].

### 4.5. Long-Term Outcomes and Implant Survival

Mortality in our cohort (34.1% at a median follow-up of 98.1 months [63.5–153.5]) was comparable to the long-term survival rates reported in similar studies of elderly patients after hemiarthroplasty or conversion procedures [4,6]. The long-term follow-up and the low incidence of late mechanical complications suggest good stability of the reconstructive technique and support the potential durability of acetabular fixation in this patient cohort.

### 4.6. Strengths and Limitations

The strengths of this study include a long median follow-up, systematic microbiological sampling, and a homogeneous surgical technique, allowing a possible assessment of infection-related erosion. The main limitations of this study include its retrospective design and the relatively small sample size, which reflects the selective inclusion of patients with available microbiological data and may have led to underrepresentation of other potentially relevant variables such as mechanical or anatomical factors. Furthermore, the retrospective nature introduces a risk of selection bias and limits the ability to control for confounding factors. The possibility of culture contamination cannot be completely excluded, despite adherence to standard microbiological procedures. Variability in follow-up duration among patients may also influence the detection of late complications. Finally, as with all observational retrospective studies, causal relationships cannot be definitively established.

## 5. Conclusions

In conclusion, early acetabular erosion following HA may raise suspicion for previously unrecognized low-grade infection, particularly in frail elderly patients. In our cohort, conversion to THA was associated with functional improvement and acceptable complication rates, suggesting that this procedure can be a reasonable option for selected symptomatic patients, even in the context of advanced age and comorbidities. Conservative antibiotic treatment appeared satisfactory in the infected cases, although the small sample limits definitive interpretation. Future prospective multicenter studies are needed to better clarify the relationship between low-grade infection, frailty, and the progression of acetabular cartilage damage.

## Figures and Tables

**Table 1:** Base line characteristics.

	Total (n = 44)
**Age at neck fracture, years**	80.5 (74.0–85.0)
**Sex, female**	35 (79.6%)
**Side, left**	26 (59.1%)
**BMI ^1^, kg/m^2^**	26 (25–26)
**FAC ^2^**	5 (4–5)
FAC 5	28 (63.6%)
FAC 4	13 (29.6%)
FAC 3	3 (6.8%)
FAC 2	0 (0.0%)
FAC 1	0 (0.0%)
FAC 0	0 (0.0%)
**Barthel Index**	95.0 (85.0–95.0)
**BADL ^3^, yes**	30 (68.18%)
**Pfeiffer scale**	2.0 (1.0–2.0)
**RCMDS ^4,5^**	0.37 ± 0.78
**Hypertension, yes**	28 (65.1%)
**Diabetes Mellitus, yes**	6 (13.6%)
**Chronic kidney disease, yes**	6 (13.6%)
**Rheumatoid arthritis, yes**	1 (2.3%)
**Immunosuppression, yes**	4 (9.1%)
Pharmacological	3
Disease-related (VIH/AIDS)	1
**Polypharmacy, yes**	27 (61.36%)

Categorical variables were expressed as counts (%), and continuous variables as median and interquartile range (IQR, P25–P75). ^1^ BMI: Body Mass Index, ^2^ FAC: Functional Ambulation Classification, ^3^ BADL: basic activities of daily living, ^4^ Red Cross Mental Disability Scale, ^5^ Despite asymmetrical data distribution, descriptive values were expressed as mean ± standard deviation to provide additional information since median [IQR] were 0 (0) in both groups.

**Table 2:** Characteristics of hemiarthroplasty.

	Total (n = 44)
**Femoral stem model**	
CS-plus^®^ (Smith & Nephew, London, UK)	21 (47.7%)
SL-plus^®^ (Smith & Nephew, London, UK)	19 (43.2%)
VerSys^®^ (Zimmer Biomet, Warsaw, IN, USA)	2 (4.6%)
Thompson^®^ (JRI Orthopaedics, Sheffield, UK)	1 (2.3%)
Exeter^®^ (Stryker, Kalamazoo, MI, USA)	1 (2.3%)
**Femoral fixation method**	
Cemented	25 (56.8%)
Press-fit	19 (43.2%)
**Head side, mm**	46 (44–46)
42	3 (6.8%)
44	10 (22.8%)
46	22 (50.0%)
48	7 (15.9%)
52	2 (4.6%)
**Monopolar**	34 (77.3%)
**Bipolar**	10 (22.7%)

Categorical variables were expressed as counts (%), and continuous variables as median and interquartile range (IQR, P25–P75).

**Table 3:** Variables related to acetabular reconstruction.

	Total (n = 44)
**Age at conversion surgery, years**	81.8 (77.5–88.8)
**Time acetabular erosion, months**	25.4 (10.4–47.4)
**Time conversion surgery, months**	43.2 (20.5–61.2)
**Approach**	
Lateral	10 (22.7%)
Posterolateral	34 (77.3%)
**Paprosky classification**	
2A	12 (27.3%)
2B	6 (13.6%)
2C	1 (2.3%)
3A	21 (47.7%)
3B	4 (9.1%)
**Bone Impaction Grafting**	29 (65.9%)
Mesh	7 (15.9%)
Tantalum supplement	1 (2.3%)
**Cup size, mm**	50 (48–50)
46	4 (9.1%)
48	17 (38.6%)
50	16 (36.3%)
52	4 (9.1%)
54	1 (2.3%)
56	1 (2.3%)
58	1 (2.3%)
**Cup fixation method**	
Cemented	31 (70.5%)
Press-fit	13 (29.5%)
**Cup model**	
Rimfit^®^ (Stryker, Kalamazoo, MI, USA)	23 (52.3%)
Contemporary^®^ (Stryker, Kalamazoo, MI, USA)	5 (11.4%)
Avantage^® 1^ (Zimmer Biomet, Warsaw, IN, USA)	3 (6.8%)
Trilogy^® 2^ (Zimmer Biomet, Warsaw, IN, USA)	3 (6.8%)
DELTA^®^ (LimaCorporate, San Daniele del Friuli, Italy)	2 (4.6%)
G7^®^ (Zimmer Biomet, Warsaw, IN, USA)	2 (4.6%)
Pinnacle^®^ (DePuy Synthes, Warsaw, IN, USA)	2 (4.6%)
Continuum^®^ (Zimmer Biomet, Warsaw, IN, USA)	3 (6.8%)
Trident^®^ (Stryker, Kalamazoo, MI, USA)	1 (2.3%)
**Screws**	10 (22.7%)
**Dual mobility**	4 (9.1%)
**Head size, mm**	
22	5 (11.3%)
28	31 (70.5%)
32	8 (18.2%)
**Intraoperative complications**	2 (4.6%)
**Follow-up, months**	98.1 (63.5–153.5)

Categorical variables were expressed as counts (%), and continuous variables as median and interquartile range (IQR, P25–P75). ^1^ Cemented, ^2^ Cementless.

**Table 4:** Baseline characteristics, hemiarthroplasty type, and their association with infection.

	Non-Infection (n = 38)	Unexpected Infection (n = 6)	*p*-Value
**Age at neck fracture, years**	82.0 (75.0–86.0)	78.5 (70.0–81.0)	0.178
**Sex**			0.089
Female	32 (72.7%)	3 (6.8%)	
Male	6 (13.6%)	3 (6.8%)	
**Side, left**			0.208
Left	24 (54.5%)	2 (4.6%)	
Right	14 (31.8%)	4 (9.1%)	
**BMI ^1^, kg/m^2^**	26.0 (25.0–26.0)	25.0 (25.0–30.0)	0.683
**FAC ^2^**	5.0 (4.0–5.0)	5.0 (4.0–5.0)	1.000
**Barthel Index**	95.0 (85.0–95.0)	95.0 (80.0–95.0)	0.717
**BADL ^3^, yes**	26 (50.1%)	4 (9.1%)	1.00
**Pfeiffer scale**	2.0 (1.0–2.0)	1.0 (1.0–2.0)	0.646
**RCMDS ^4,5^**	0.4 ± 0.82	0.17 ± 0.41	0.854
**Hypertension, yes**	24 (54.5%)	4 (9.1%)	1.000
**Diabetes Mellitus, yes**	4 (9.1%)	2 (4.6%)	0.182
**Chronic kidney disease, yes**	4 (9.1%)	2 (4.6%)	0.182
**Rheumatoid arthritis, yes**	1 (2.3%)	0 (0.0%)	1.000
**Immunosuppression, yes**	3 (6.8%)	1 (2.3%)	0.456
**Polypharmacy, yes**	23 (52.3%)	4 (9.1%)	1.000
**Femoral stem model**			0.247
CS-plus^®^ (Smith & Nephew, London, UK)	16 (36.4%)	5 (11.4%)	
SL-plus^®^ (Smith & Nephew, London, UK)	19 (43.2%)	0 (0.0%)	
VerSys^®^ (Zimmer Biomet, Warsaw, IN, USA)	1 (2.3%)	1 (2.3%)	
Thompson^®^ (JRI Orthopaedics, Sheffield, UK)	1 (2.3%)	0 (0.0%)	
Exeter^®^ (Stryker, Kalamazoo, MI, USA)	1 (2.3%)	0 (0.0%)	
**Femoral fixation method**			0.029
Cemented	19 (43.2%)	6 (13.6%)	
Press-fit	19 (43.2%)	0 (0.0%)	
**Head side, mm**	46.0 (44.0–46.0)	46.0 (44.0–48.0)	0.876
**Monopolar**	30 (68.2%)	4 (9.1%)	1.000
**Bipolar**	8 (18.2%)	2 (4.6%)	
**Time acetabular erosion, months**	29.8 (14.8–50.7)	4.0 (3.7–4.6)	<0.001
**Time conversion surgery, months**	48.2 (21.0–63.9)	30.0 (20.0–41.3)	0.157
**Age at conversion surgery, years**	84.6 (77.8–89.6)	78.8 (70.3–81.3)	0.064
**Paprosky classification**			0.109
2A	8 (18.2%)	4 (9.1%)	
2B	6 (13.6%)	0 (0.0%)	
2C	1 (2.3%)	0 (0.0%)	
3A	20 (45.5%)	1 (2.3%)	
3B	3 (6.8%)	1 (2.3%)	
**Postoperative complications**	8 (18.2%)	1 (2.3%)	1.000

Categorical variables were expressed as counts (%), and continuous variables as median and interquartile range (IQR, P25–P75). ^1^ BMI: Body Mass Index, ^2^ FAC: Functional Ambulation Classification, ^3^ BADL: basic activities of daily living, ^4^ Red Cross Mental Disability Scale, ^5^ Despite asymmetrical data distribution, descriptive values were expressed as mean ± standard deviation to provide additional information since median (IQR) were 0 (0) in both groups.

**Table 5:** Clinical and radiological outcomes during follow-up.

	Non-Infection (n = 38)	Unexpected Infection (n = 6)	*p*-Value
**Follow-up**	99.0 (67.0–150.3)	77.3 (34.1–115.1)	0.171
**Postoperative complications**	8 (18.2%)	1 (2.3%)	1.000
**Time to complication, months**	93.0 (50.0–150.3)	55.0 (34.1–143.5)	0.274
**X-Ray analysis postoperative**			
Acetabular inclination, °^1^	42.0 (40.0–45.0)	42.5 (40.0–45.0)	0.986
Vertical distance, mm	20.0 (15.0–26.0)	29.0 (25.0–35.0)	0.080
Horizontal distance, mm	22.0 (20.0–24.0)	20.0 (20.0–24.0)	0.073
Distance to CR ^1^, mm	6.0 (2.0–17.0)	18.5 (12.0–25.0)	0.119
**X-Ray analysis last follow-up**			
Acetabular inclination, °	43.5 (40.0–48.0)	41 (40.0–45.0)	0.809
Vertical distance, mm	15.5 (12.0–20.0)	15.0 (10.0–30.0)	0.889
Horizontal distance, mm	30.0 (28.0–30.0)	32.5 (27.0–35.0)	0.449
Distance to CR ^2^, mm	3.0 (2.0–8.0)	10.0 (3.0–12.0)	0.162
**mHHS ^3^ preoperative**	48.0 (46.0–50.0)	46.0 (46.0–50.0)	0.647
**mHHS ^3^ last follow-up**	89.0 (84.0–90.0)	87.0 (86.0–90.0)	0.529
**Mortality during follow-up**	12 (12.3%)	3 (6.8%)	0.394
**Time to death, months**	97.2 (67.4–142.8)	42.0 (34.1–115.8)	0.248

Categorical variables were expressed as counts (%), and continuous variables as median and interquartile range [IQR, P25–P75]. ^1^ °: degrees. ^2^ CR: centre of rotation. ^3^ mHHS: modified Harris Hip Score.

## Data Availability

Data are unavailable due to ethical and privacy restrictions related to clinical practice.
